# Self-Organized Vascularized Hepatic Organoids in Microcapsules for Liver Regeneration

**DOI:** 10.34133/research.0898

**Published:** 2025-09-19

**Authors:** Yue Zhi, Jinglin Wang, Danqing Huang, Yuanjin Zhao

**Affiliations:** ^1^ Department of Rheumatology and Immunology, Nanjing Drum Tower Hospital, Clinical Medical College of Traditional Chinese and Western Medicine, Nanjing University of Chinese Medicine, Nanjing 210009, China.; ^2^ School of Biological Science and Medical Engineering, Southeast University, Nanjing 210096, China.

## Abstract

Hepatic organoids represent a promising strategy for liver regeneration; however, challenges remain in flexibly tailoring them to achieve complex architectures and vascularized structures for enhanced functionality. Here, we propose a novel system of self-organized vascularized hepatic organoids (SOVHOs) encapsulated in hydrogel microcapsules with biomimetic features for liver regeneration. The hydrogel shell microcapsules encapsulating human umbilical vein endothelial cells and hepatic organoids derived from human induced pluripotent stem cells were generated using microfluidic encapsulation technology. The hydrogel shell provides a 3-dimensional culture environment that supports the formation of uniform hepatocyte spheroids with vascular networks inside the microcapsules. The SOVHOs exhibit liver-specific functions, including drug metabolism, urea synthesis, and serum protein production. In rats with acute liver failure, we demonstrated that the SOVHOs markedly enhanced survival rate and normalize the inflammatory response after transplantation, indicating their remarkable repopulation capacity in facilitating injured liver recovery. Furthermore, SOVHOs remained viable in the host liver for at least 7 days post-implantation, exhibiting low immunogenicity and no detectable adverse effects in rats during this period. These results suggest that our SOVHOs are potentially valuable for clinical application in liver disease treatment.

## Introduction

The regeneration process, which is vital for the recovery of liver injury, can be compromised by numerous factors [1,2]. Among them, temporarily improving essential hepatic metabolic functions is crucial for prolonging the patient’s life or regenerating the hepatic tissue until a donor liver becomes available [3,4]. Nowadays, a variety of methods have been developed for this purpose, including stem cell therapy [5], extracorporeal liver support [6], and hepatocyte transplantation [7–10]. Among these strategies, stem cell therapy has shown promising advantages and practical feasibility [11–13]. Notably, integrating with advanced materials and processing technologies that support stem cell growth and differentiation can enhance their therapeutic efficiency [14–18]. Specifically, considerable effort has been directed toward hydrogel substrates and microfluidic encapsulation to regulate stem cell fate and improve cell viability [19–25]. Despite the huge and continuing advances in stem cell therapy, some challenges still hinder its clinical translation, such as selecting the ideal cell type, maintaining liver physiology, and ensuring long-term function and safety after transplantation [26]. Therefore, it is anticipated to develop new strategies to facilitate liver regeneration.

**Fig. 1.**
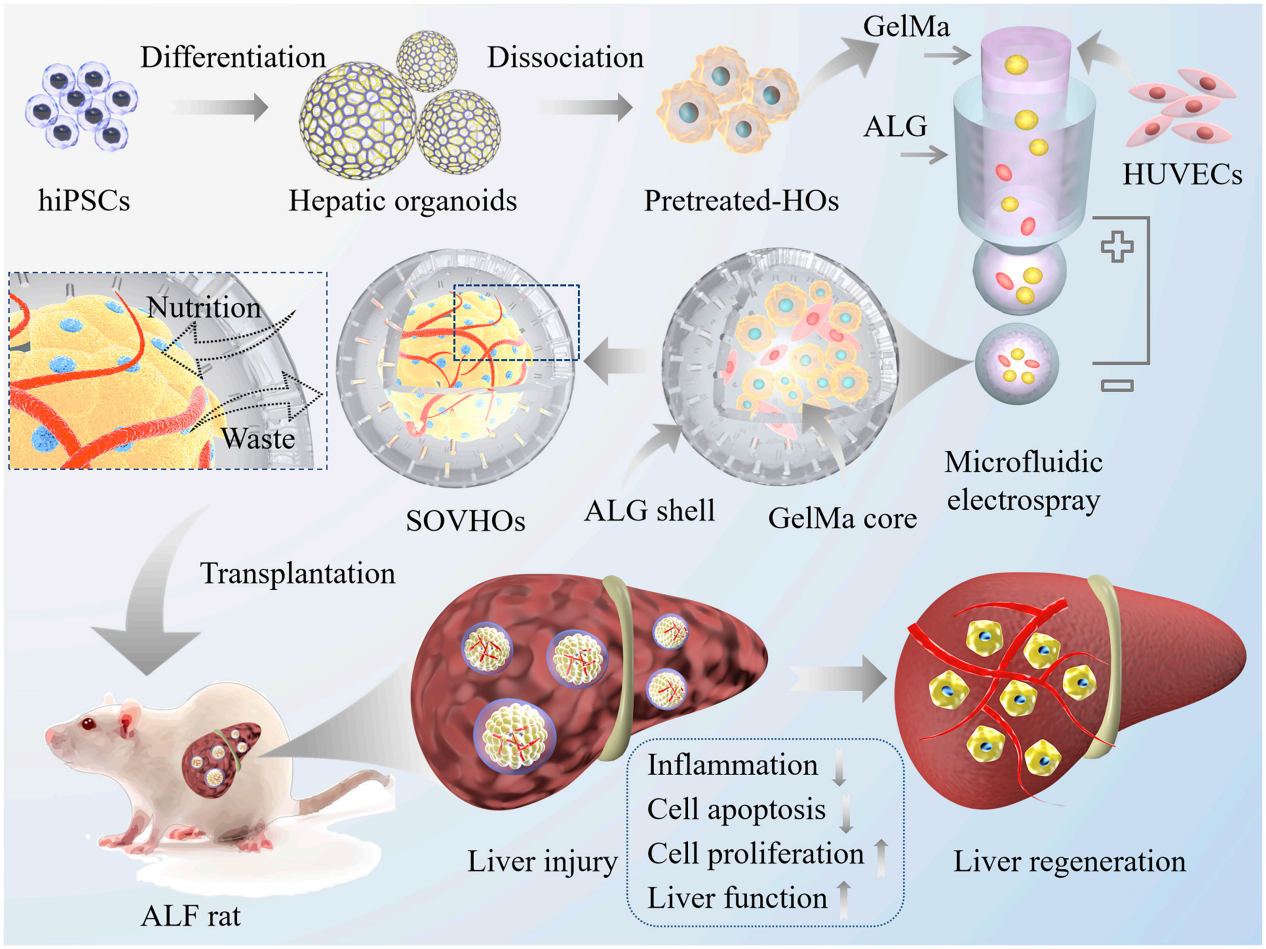
Schematic illustration of self-organized vascularized hepatic organoids integrated with microcapsules. Dissociated hepatic organoids and endothelial cells were encapsulated within microcapsules via microfluidic electrospray. This innovative 3-dimensional (3D) culture method produced differentiated mature hepatic organoids with intricate vascular networks. Furthermore, this structure was injectable and capable of being implanted in situ into ALF rats, thereby facilitating the repair of damaged liver tissue.

Here, we developed a novel system of self-organized vascularized hepatic organoids (SOVHOs) with essential biomimetic characteristics utilizing microfluidic hydrogel microcapsules for liver repair, as illustrated in Fig. 1. Organoids have emerged as a powerful and revolutionary biotechnology in recent years [27–32]. Among them, hepatic organoids (HOs) play vital roles in liver biology exploration [33,34], drug discovery [35–37], and liver failure treatments [38,39]. Specifically, human induced pluripotent stem cells (hiPSCs) are an ideal source for the construction of HOs, allowing inexhaustible production and substantially reduced immune rejection for liver transplantations [40–43]. Although several organoid generation techniques have emerged, few of these can control the size and shape of cell structures and replicate cellular diversity and complex cell–cell interactions [44,45]. In addition, since the exchange of nutrients and wastes is necessary for long-term cultivation and practical applications, it is difficult for current technologies to construct highly reproducible vascularized organoids with perfusable endothelial networks [46]. In contrast, our SOVHOs in microcapsules could potentially offer a promising solution to address several current issues and may be beneficial for liver regeneration.

In this paper, we generate the size-controllable HOs with vascularized structures to support cell proliferation and self-condensation and promote liver maturation by using microfluidic encapsulation technology. Based on this technology, hydrogel shell microcapsules encapsulating HO and endothelial cells in the core were formed. The hydrogel shell provided structural support to the cells, enabling them to self-organize and differentiate into hepatocyte spheroids with endothelial networks and making their size controllable. It was demonstrated that these vascularized HOs could realize nutrients and gas exchange required for long-term cell culture. Additionally, the SOVHOs in microcapsules exhibited properties of mature hepatocytes, such as increased albumin secretion, urea production, and other important parameters of hepatocyte-specific functions. To elucidate the practicability of our SOVHOs for liver regeneration, we employed them in the treatment of rats with acute liver failure (ALF). Results indicated that the SOVHOs could respond to systemic regenerative cues after implantation, facilitating injury recovery and liver regeneration. Moreover, the encapsulated human umbilical vein endothelial cells (HUVECs) network provided immune protection and facilitates nutrient and oxygen delivery, enabling the survival of our SOVHOs in the host liver. Therefore, our SOVHOs have shown great promise for liver regeneration and have proven valuable for future clinical research on liver failure.

## Results

### Differentiation of hiPSCs into HOs

**Fig. 2.**
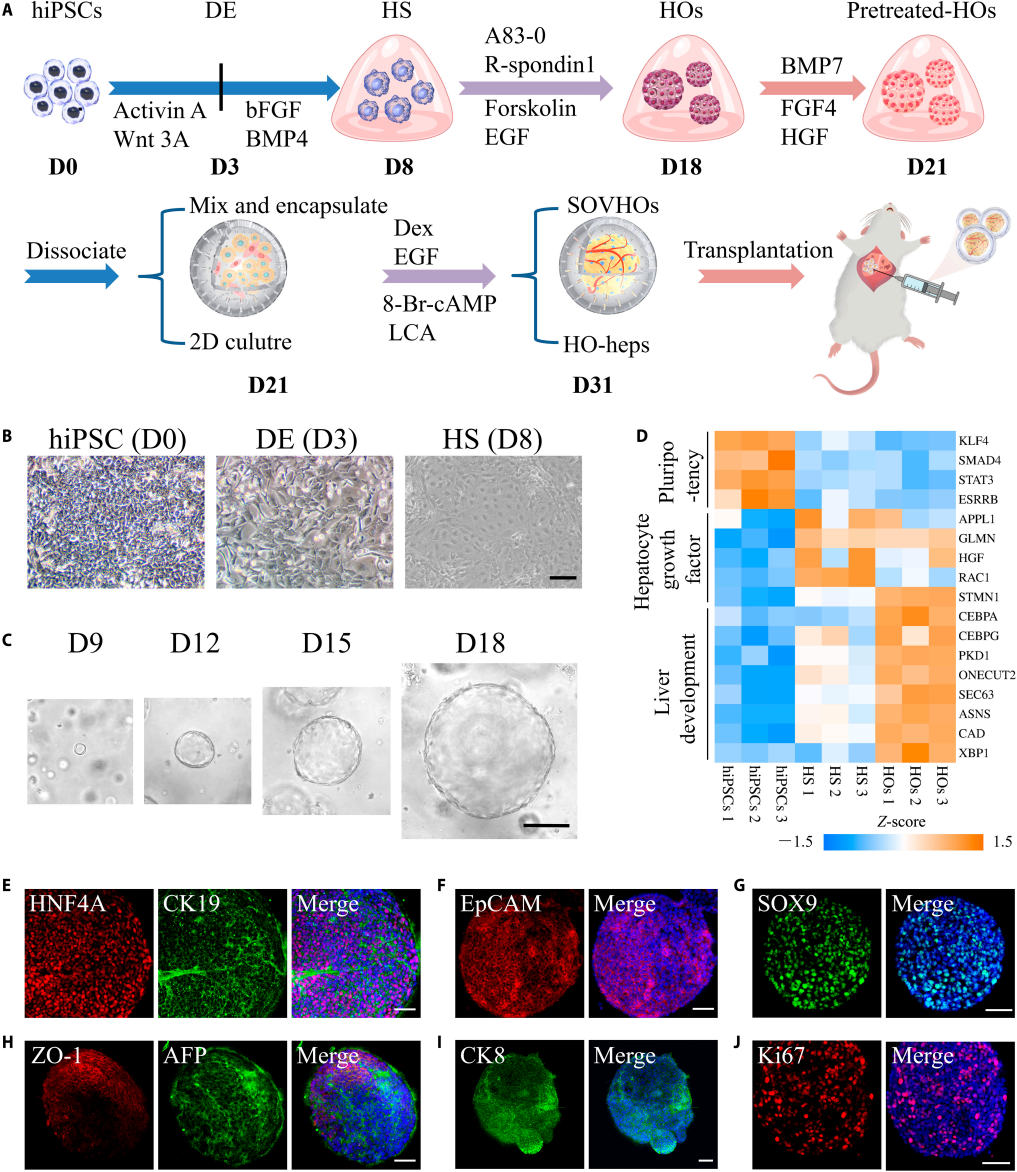
Characterization of differentiated cells derived from hiPSCs at different differentiation stages. (A) Schematic of the experimental design for generating SOVHOs. (B and C) Representative pictures of HOs cultures at various stages of development. (D) Heatmap exhibiting the expression levels of specified genes about pluripotency, hepatocyte growth factor, liver development in hiPSCs, HS cells, and HOs. (E to J) Representative immunofluorescence images of HOs stained with specific antibodies for various hepatic stem/progenitor markers. Scale bar, 100 μm.

The stepwise differentiation from hiPSCs to HOs has been shown in Fig. 2A to C, utilizing previously described protocols that have been modified [47]. We initially generated hepatic endoderm cells from hiPSCs through directed differentiation. The definitive endoderm (DE) cells (day 3) were subsequently induced into hepatic specification (HS) cells (day 8). Then, the resultant cells were identified to determine if they expressed liver progenitor cell markers. The expression of pluripotency markers was decreased, coinciding with an increase in EpCAM, CK19, and SOX9 expression in the HS group, as demonstrated by quantitative real-time PCR (qPCR) (Fig. S1). HOs initially adopted a hollow spherical morphology and rapidly expanded into substantial 3-dimensional (3D) structures within 10 days. To investigate the molecular characteristics of HOs, we performed RNA sequencing on cells at various developmental stages. This analysis included the transcriptomes of multiple cell types, including hiPSCs, HS cells, and HOs. Principal component analysis revealed distinct clustering among the analyzed groups (Fig. S2A). Additionally, the heatmap indicated that hiPSCs exhibited high expression of specific pluripotency markers. The HS group demonstrated up-regulation of multiple hepatocyte growth factors, while HOs exhibited increased expression of Wnt signaling components and regulators of liver development (Fig. 2D and Fig. S2B). The difference between HS cells and HOs was confirmed by qPCR, revealing unregulated genes of early liver development in the HS group, alongside the up-regulation of genes associated with hepatic stem/progenitor cells in HOs (Fig. S3). Immunofluorescence assays on day 18 demonstrated the expression of hepatic stem/progenitor cell markers in HOs (Fig. 2E to I). Furthermore, Ki67 immunofluorescence assays indicated cell proliferation within HOs (Fig. 2J). These findings suggested that such sequential treatments facilitate the effective differentiation of hiPSCs into HOs.

### Coculture in microcapsules

**Fig. 3.**
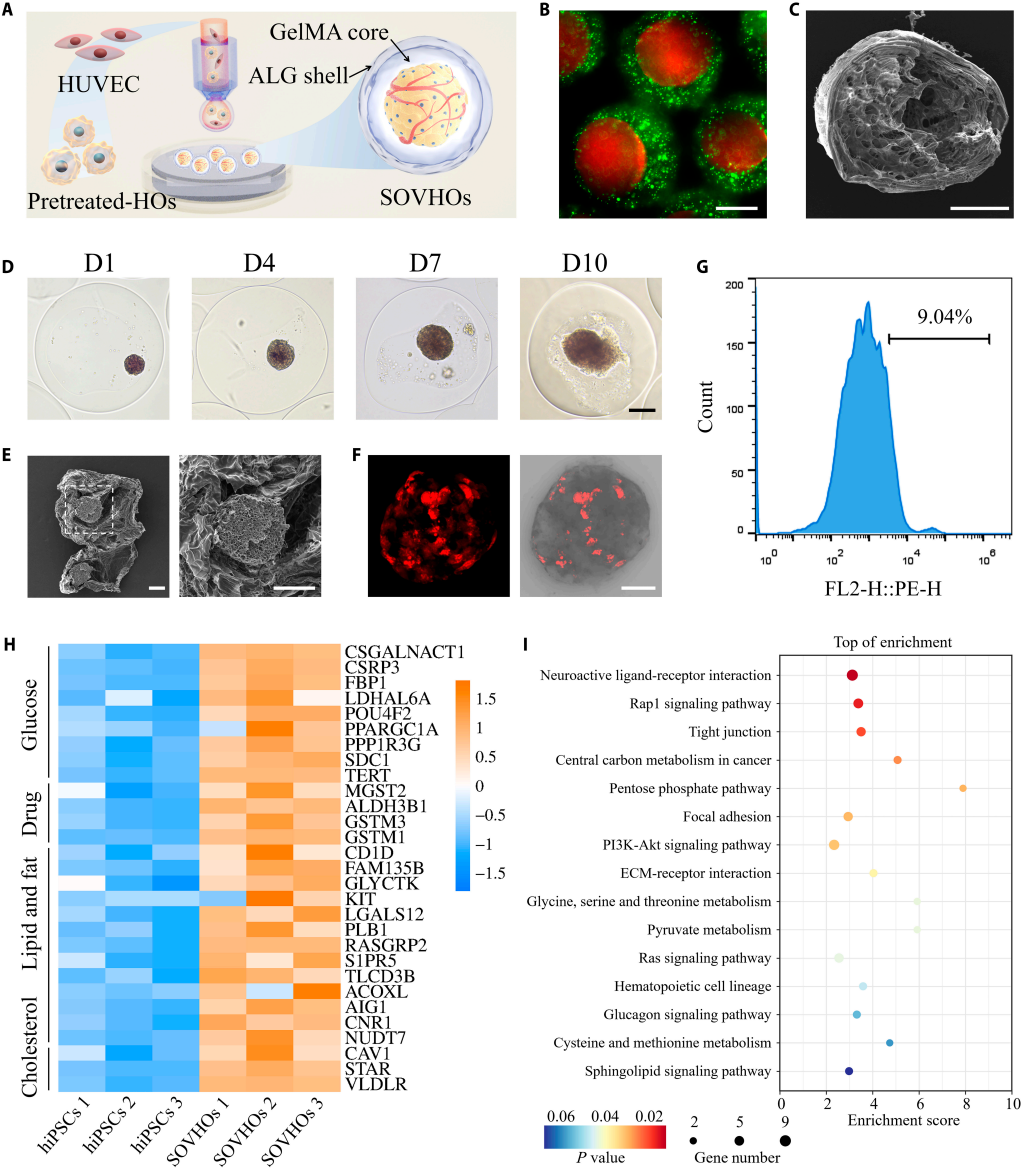
Characterization of SOVHOs within hydrogel microcapsules. (A) The schematic illustration showed the generation process of SOVHOs. (B) Fluorescent polystyrene nanoparticles were utilized to represent core and shell layers. (C) SEM image revealed the core–shell structure. (D) Representative images of SOVHOs were captured throughout a 10-day culturing period. (E) SEM image showing the whole SOVHOs and the magnified image (3×) showing the surface structure. (F) Representative images of HUVECs (staining with cherry lentivirus, red fluorescence) cocultured with hepatocytes derived from hepatic organoids in microcapsules via a confocal scanning microscope. (G) The viability of SOVHO was evaluated using flow cytometry. (H) Heatmap illustrated gene expression associated with specific hepatic functions, such as drug detoxification, lipid metabolism, and cholesterol synthesis. (I) Bubble chart presented the results of differentially expressed genes through KEGG pathway enrichment. Scale bars, 200 μm (B and C), 100 μm (D and E), and 50 μm (F).

Hepatocytes cocultured with HUVECs have demonstrated the ability to maintain their viability and distinctive liver-like characteristics. The coculture conditions facilitated exposure of hepatocytes to paracrine signals generated by HUVECs, along with signals arising from cell–cell interactions, which closely resemble the cellular processes involved in liver organogenesis. These findings suggest that HUVECs are instrumental in the development of 3D liver tissue [48]. To reproduce bionic hepatic tissue with vascular networks in vitro, we constructed a microphysiological cell culture system for SOVHOs in microcapsules through microfluidic electrospray technology (Fig. 3A). Initially, HOs (day 18) were pretreated for 3 days with Advanced DMEM/F12, supplemented with BMP7, FGF4, and HGF. Following this, the pretreated HOs (day 21) were dissociated into single cells. We utilized sodium alginate (ALG) as outer phase while HUVECs and dissociated pretreated HOs (day 21)-dispersed gelatin methacrylamide (GelMA) solution was used as inner phase and encapsulated using microfluidic technology. The resulting cell mixture in microcapsules was then cultured for 10 days to generate the SOVHOs (day 31). GelMA with high biocompatibility has been demonstrated to enhance cellular physiology and behavior, and ALG is a widely applied biosafe material for tissue engineering. Microcapsules composed of ALG shell and non-crosslinked GelMA core encapsulating cells can be fabricated through microfluidic electrospray technology in a single step. The non-crosslinked GelMA core preserved a soft and permissive microenvironment that supported rapid cell–cell interaction and spheroidization and allowed dynamic cell remodeling and migration, which were essential for vascular network formation and hepatic maturation. Red and green fluorescent nanoparticles labeled the GelMA and ALG components, respectively, enabling the visualization of the microcapsules’ core–shell structure (Fig. 3B). Scanning electron microscopy (SEM) was employed to further characterize freeze-dried microcapsules, which revealed that the microcapsules exhibited core–shell structure and heterogeneous pores (Fig. 3C). The results indicate that the diameter of the microcapsules increases with the collection distance, while conversely, it decreases as the applied voltage increases (Fig. S4A and B). Through dynamic adjustment of the flow rate, collection distance, and voltage, we optimized the conditions with an inner flow rate (*F*_inner_) of 10 μl min^−1^, an outer flow rate (*F*_outer_) of 50 μl min^−1^, a voltage of 6 kV, and a collection height of 4 cm. Under these conditions, the resulting microspheres exhibited a diameter of approximately 440 μm and a nucleus diameter of approximately 370 μm, with exceptional monodispersity, as demonstrated in Fig. S4B and C. Furthermore, fluorescent compounds of varying molecular weights were utilized to characterize the permeability of the microcapsules. Over a 60-min period, the fluorescence intensity within the core exhibited a gradual decline, indicating that these microcapsules possess high permeability, thereby facilitating the transport of nutrients and oxygen during cellular growth (Fig. S5A). As illustrated in Fig. S5B, ALG exhibits no significant change in mass over a 10-day period. Furthermore, after immersion in the culture medium for 5 and 10 days, ALG retained its mechanical properties (Fig. S5C). These results suggested that the hydrogel maintains mechanical integrity over extended periods.

The 3D structure of microcapsules allows for high-throughput cell encapsulation and guarantees consistent cell aggregate size, and the cellular microenvironment it creates can facilitate the formation of bionic-sized human tissue analogs. It was found that the ALG shell provided a 3D culture environment for HOs to differentiate into mature hepatocytes without any potential toxicity to the cells. After encapsulation, dispersed pretreated-HOs and HUVECs underwent spontaneous aggregation, driven by cell–cell adhesion and self-organization within 6 h (Fig. S6A). After 10-day culture, spontaneous aggregation of cells into uniformly sized solid spheroids with smooth edges was observed (Fig. 3D and Fig. S6B and C). The surface characteristics and structure of SOVHOs were validated through SEM imaging, as demonstrated in Fig. 3E. Moreover, transfected HUVECs were identified through red fluorescence using the cherry lentiviral vector. Confocal microscopy analysis, as depicted in Fig. 3F, confirmed the even distribution of HUVECs throughout the hepatocytes derived from HOs. Cell viability of the SOVHOs was assessed using flow cytometry, with results indicating a high proportion of liver cells following microencapsulation (Fig. 3G), and viability assay was routinely performed at 4, 7, and 10 days, displaying >90% live cells using calcein acetoxymethyl ester (Calcein AM) and propidium iodide (PI) (Fig. S6D and E).

To enhance our understanding of the differentiation and maturation processes of SOVHOs, we conducted an analysis of the expression patterns of genes characteristic of the mature liver by transcriptomic sequencing. Notably, RNA sequencing analysis showed that genes encoding proteins and enzymes essential for various hepatic functions, such as drug detoxification, lipid metabolism, and cholesterol synthesis, exhibited significant up-regulation in the SOVHOs group, as illustrated in Fig. 3H. Simultaneously, the major signaling pathways involved were investigated by Kyoto Encyclopedia of Genes and Genomes (KEGG) pathway enrichment analysis. The results revealed substantial enrichment of differentially expressed genes within extracellular matrix (ECM)–receptor interaction, P13K-Akt signaling pathway, and pentose phosphate pathway, all of which are closely linked to liver homeostasis and hepatic differentiation (Fig. 3I). To validate the transcriptomic findings and further confirm the hepatic maturation of SOVHOs, we performed qPCR analysis on a selected panel of liver-specific genes including CYP3A4, ALB, and HNF4A and confirmed a significant up-regulation of these hepatic genes in SOVHOs compared to hiPSCs (Fig. S7).

### Function assessment

**Fig. 4.**
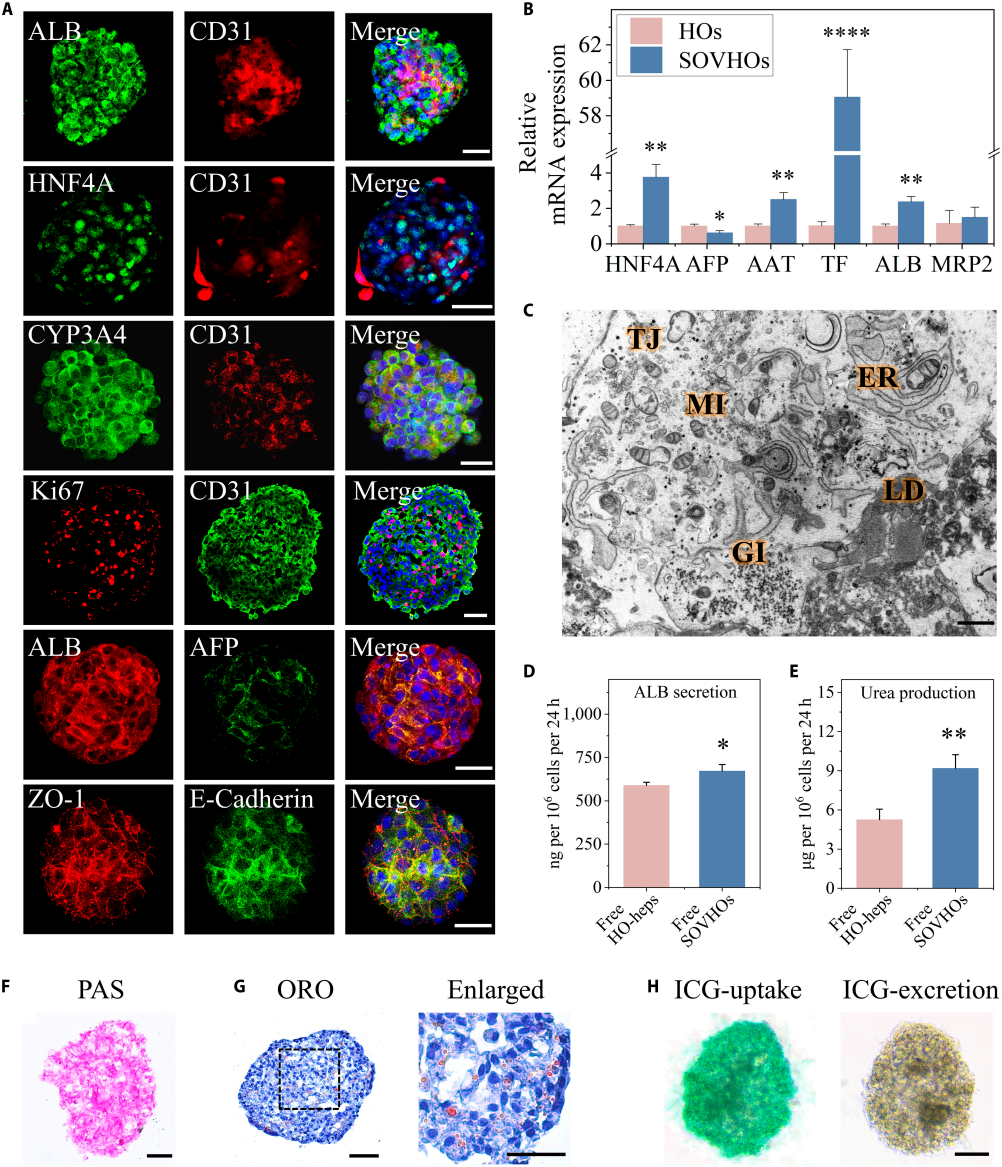
Functional identification of vascularized hepatocytes. (A) Immunostaining revealed that SOVHOs expressed the endothelial marker CD31, along with several hepatocyte markers indicative of polarity and maturation. (B) Hepatic lineage markers were analyzed in HOs and SOVHOs using qPCR (*n* = 3 per group). (C) Ultrastructure of SOVHOs demonstrated the morphological features of mature hepatocytes. ER, endoplasmic reticulum; Mi, mitochondria; LD, lipid droplet; TJ, tight junction; Gl, glycogen. (D and E) ELISAs were conducted to quantify the levels of ALB and urea in the culture supernatants of SOVHOs and HO-heps (*n* = 3 per group). (F) Glycogen storage within SOVHOs was visualized using PAS staining. (G) ORO staining showed the accumulated liquid in SOVHOs. (H) ICG excretion test of SOVHOs at the beginning and after 6 h of culturation. Scale bars, 50 μm (A and F to H) and 1 μm (C). *P < 0.05, **P < 0.01, ****P < 0.0001.

Immunostaining for ALB, CYP3A4, HNF4A, AFP, and CD31 was conducted to investigate the morphological and functional characteristics of self-organized 3D vascularized HO-derived hepatocyte spheroids (Fig. 4A). Notably, E-cadherin protein was found to be abundant in the SOVHOs, suggesting the deposition of extracellular matrix components. Additionally, we evaluated the expression of several key functional genes within the composite spheroids. As shown in Fig. 4B and Fig. S8, qPCR analysis revealed that the expression of genes associated with liver-specific functions, urea cycle enzymes, and drug metabolism was significantly increased in SOVHOs at day 31 when compared with HOs at day 18. Flow cytometric characterization revealed that approximately 11.8% of SOVHOs expressed AFP, 70% expressed ALB, and 11.5% expressed both ALB and AFP (Fig. S9). When imaged with a transmission electron microscope, the distinctive ultrastructure of polarized hepatocytes is evidenced in the SOVHOs, where bile ducts are observed situated between the apical membranes (Fig. S10). Additionally, according to transmission electron microscopy, the SOVHOs were abundant in mitochondria and endoplasmic reticulum, displaying lipid droplet accumulation and glycogen storage akin to those of mature hepatocytes (Fig. 4C). In comparison to free hepatocytes derived from HOs (free HO-heps), SOVHOs demonstrated significantly enhanced levels of ALB secretion and urea production by enzyme-linked immunosorbent assays (ELISAs) (Fig. 4D and E). Then, series of assays were conducted to elucidate the functional roles of SOVHOs. The glycogen storage capacity of SOVHOs was confirmed through periodic acid–Schiff (PAS) staining (Fig. 4F). Additionally, the aggregation of lipid droplets revealed by Oil Red O (ORO) staining indicated the lipid metabolic capabilities of these hepatocyte-like cells derived from HOs (Fig. 4G). Furthermore, SOVHOs exhibited the ability to both absorb and release indocyanine green (ICG) (Fig. 4H). Collectively, these findings provide compelling evidence that SOVHOs are capable of executing specific liver functions, thereby suggesting their substantial therapeutic potential in ALF.

### In vivo therapeutic efficiency

**Fig. 5.**
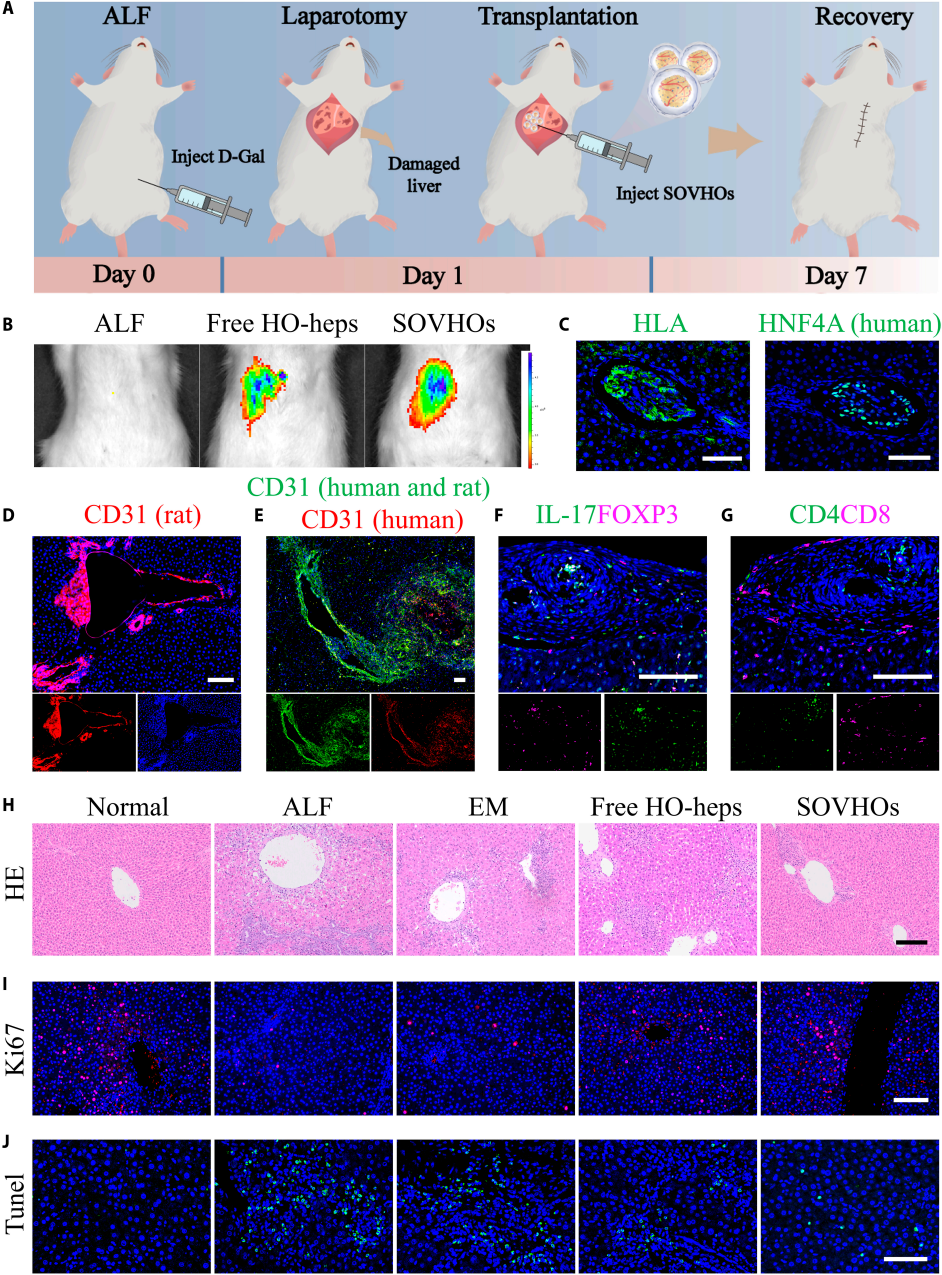
In vivo experiments assessing the effects of SOVHOs in curing ALF rats. (A) Schematic diagram of SOVHOs transplantation into rat with D-Gal-induced liver failure. (B) Fluorescence images to confirm DiD-stained SOVHOs in the liver after transplantation. (C to E) Immunofluorescent analysis of HLA, human HNF4A, and CD31 expression in transplants on day 7. (F and G) Immunohistochemical staining for IL-17, FOXP3, CD4, and CD8 was performed in all groups at day 7 following SOVHOs transplantation. (H) H&E staining of liver tissues after transplantation. (I and J) Staining of markers Ki67 and TUNEL in all groups. Scale bar, 100 μm.

The utilization of encapsulated hepatocyte transplantation has become a prevalent approach for providing temporary liver function support [49]. The implantation efficiency and metabolic stability have been improved through the encapsulation of either patterned or randomly organized endothelial cells [50]. With access to large numbers of SOVHOs using microfluidic technology, we assessed whether these spheroids could be transplanted and treat liver failure, as schemed in Fig. 5A. Rats were administrated with D-galactosamine (D-Gal) after fasting overnight to establish the ALF rat model, and SOVHOs (containing 5 × 10^6^ hepatocytes) were transplanted in situ into the hepatic region of the rats 24 h later (Fig. S11). Healthy rats without any treatment were set as the normal group. The ALF rats were randomly assigned to 4 groups: ALF rats without treatment (ALF group), ALF rats treated with empty microcapsules (EM group), ALF rats treated with free hepatocytes derived from HOs (free HO-heps group), and ALF rats treated with SOVHOs (SOVHOs group). Successful in vivo implantation of DiD-labeled SOVHOs was evidenced by visible fluorescence in the liver lobes (Fig. 5B).

The 3D microcapsules provide immunoprotection and a semipermeable barrier for the encapsulated cells. This barrier facilitates the delivery of nutrients and oxygen while allowing for the release of secreted proteins, to optimize cellular activity and ensure cell viability. We employed immunofluorescence labeling on sections of graft/host tissue to assess the presence and preservation of various hepatocyte cell types post-transplantation. As illustrated in Fig. 5C, HLA-positive and human HNF4A-positive cells can be observed within the graft material. Notably, human CD31-positive cells from SOVHOs were found entering and extending in the rat liver, indicating that the aggregated and stacked SOVHOs might induce functional 3D structures such as microvessels, potentially establishing connections between the human-derived vascular network and the host vascular system (Fig. 5D and E). Moreover, surrounding the transplanted SOVHOs, immunohistochemical analysis of immune cell infiltration revealed a relatively low amount of IL-17^+^, FOXP3^+^, CD4^+^, and CD8^+^ cells. These findings suggest that SOVHOs exhibit low immunogenicity, eliciting only a mild host immune response (Fig. 5F and G). This might be attributed to both the biophysical protection provided by the ALG capsule and the relatively low immunogenicity of the encapsulated cell types, particularly HUVECs, which contribute to the minimal immune infiltration observed [38,51]. We have conducted extended in vivo studies before and observed that rats receiving SOVHO transplantation exhibit prolonged survival. Specifically, animals were monitored for up to 30 days post-transplantation, during which time they remained viable, with no signs of acute rejection or liver failure. Liver injury and recovery of ALF rats from different groups was further evaluated by histopathological analyses. In contrast with ALF and EM groups, ALF rats treated with SOVHOs exhibited minimal necrosis at day 7, proving the efficient therapeutic efficacy (Fig. 5H). Besides, other major organs, stained with hematoxylin–eosin (H&E), did not display significant tumorigenicity or inflammation (Fig. S12). SOVHOs exhibited elevated levels of hepatoprotection and regenerative capacity, as indicated by Ki67 staining and terminal deoxynucleotidyl transferase dUTP nick-end labeling (TUNEL) immunofluorescence analyses, which demonstrated their efficacy in promoting hepatocyte proliferation while inhibiting apoptosis (Fig. 5I and J and Fig. S13).

**Fig. 6.**
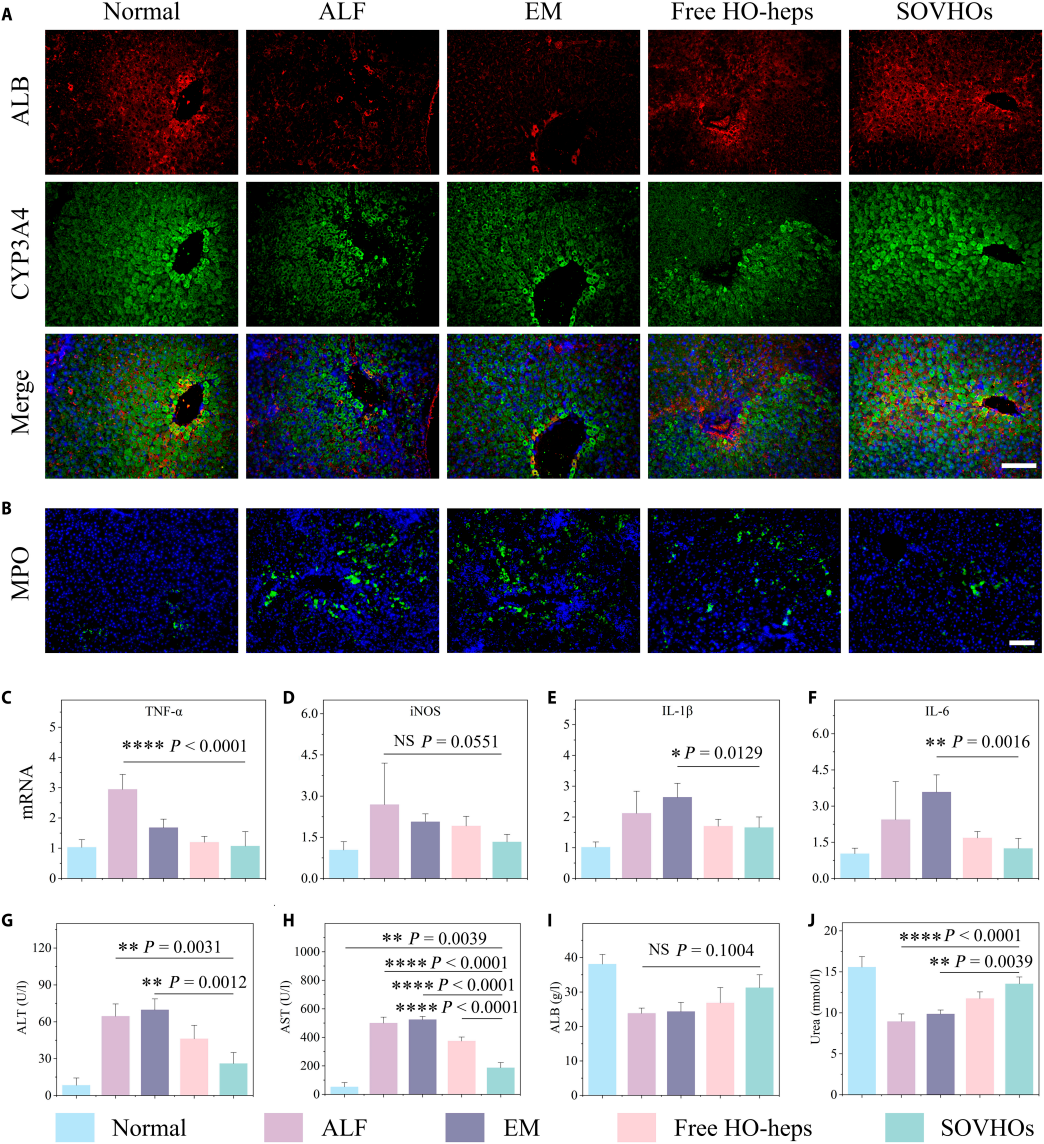
SOVHOs treatment improved liver function in groups with ALF. (A) ALB and CYP3A4 staining of liver tissues after transplantation. (B) MPO immunostaining of liver histology in all groups on day 7. (C to F) Relative mRNA expression of inflammation-related genes in different groups on day 7 (*n* = 5 per group). (G to J) Serum biochemical parameters from different treatment groups, including levels of AST, ALT, ALB, and urea (*n* = 3 per group). Scale bar, 100 μm.*P < 0.05, **P < 0.01, ****P < 0.0001.

To evaluate the therapeutic potential of SOVHOs, liver tissue in different groups were evaluated by ALB and CYP3A4 immunofluorescence staining. The graft-subjected group contained more CYP3A4 and ALB double-positive cells compared to the ALF group (Fig. 6A). To evaluate the role of trained immunity in SOVHOs and its impact on inflammation, we used immunohistochemical staining to assess myeloperoxidase (MPO) neutrophils. Since increased hepatic neutrophil infiltration is recognized as an indicator of ALF, the analysis revealed a higher presence of MPO neutrophils in the ALF and EM control groups compared to the SOVHOs-treated rats, indicating that SOVHOs treatment reduced liver inflammation (Fig. 6B). In addition, by employing qPCR on liver samples from ALF rats that received transplantation of SOVHOs, we obtained a comprehensive assessment of the expression of genes associated with hepatic inflammation. Notably, key pro-inflammatory mediators and factors were down-regulated in livers treated with SOVHOs compared to those subjected to ALF and EM treatment (Fig. 6C to F). Following therapy, blood samples were collected to monitor liver failure recovery. In comparison to the ALF and EM groups, the SOVHO-treated group exhibited a substantial increase in ALB and urea levels, along with a decrease in ALT and AST (Fig. 6G to J). In summary, SOVHO therapy demonstrates therapeutic benefits in D-Gal-induced liver failure, potentially reducing hepatocellular necrosis and ultimately enhancing hepatic regeneration.

## Conclusion

In conclusion, we presented a novel way for the bulk production of 3D vascularized HO-derived hepatocytes suitable for transplantation and potential therapeutic applications in ALF. Both in vitro and in vivo assessments demonstrated that spheroids of SOVHOs maintained high viability and exhibited mature liver functions. Additionally, these SOVHOs-loaded hydrogel microcapsules can be directly administered into the injured liver. The D-Gal-induced ALF model was specifically utilized to assess the therapeutic efficacy of SOVHOs. Encapsulation of microcapsules and coculturing with HUVECs markedly enhanced therapeutic effectiveness by promoting cell survival in an inflammatory environment. These findings indicate that SOVHOs possess liver-like characteristics, markedly improve cell survival, and reduce liver damage, thereby presenting a promising hepatocyte-based therapeutic strategy for the treatment of ALF.

## Materials and Methods

Additional information about the materials and methods used for this work is available in the Supplementary Materials.

## Supplementary Materials

Supplementary 1 Experimental Section Figs. S1 to S13 Table S1
