# Supplementary material for: Self-Organized Vascularized Hepatic Organoids in Microcapsules for Liver Regeneration
Source: Research (Wash D C). 2025 Sep 19;8:0898. doi: 10.34133/research.0898 (PMC12446754; doi:10.34133/research.0898)
Supplement: Supplementary 1 — Experimental Section Figs. S1 to S13 Table S1 [file research.0898.f1.docx]

**Supplementary Information**

**Experimental Section**

**Generation and expansion of HOs from hiPSCs**

hiPSCs were the gift from the Clinic Stem Cell Center at Nanjing Drum Tower Hospital and were cultivated in mTeSR1 media on 6-well plates. Subsequently, the hiPSCs (5 × 10^5^ cells per well) were cultured in RPMI1640 media supplemented with B27 (1%), Wnt3a (50 ng/mL) and Activin A (100 ng/mL) for three days to induce into DE cells. Then, the cells were cultured for five days in hepatocyte culture media (HCM, Lonza) supplemented with BMP4 (20 ng/mL) and FGF-2 (10 ng/mL) to generate HS cells. Following the enzymatic dissociation of the cells, Matrigel was employed for embedding the cells in 24-well plates. The cells were then cultivated in DMEM/F12 medium containing HEPES (1%), glutamine (1%), B27 (1%), N2 (1%), N-acetylcysteine (1.25 mM), gastrin (10 nM), nicotinamide (10 mM), forskolin (10 μM), A83-01 (5 μM), R-spondin1 (250 ng/mL), Wnt3a (100 ng/mL), and EGF (50 ng/mL) for ten days, leading to their differentiation into HOs.

**HOs differentiated into hepatocytes and vascularized in microcapsules**

To facilitate the differentiation of HOs into mature hepatocytes, the organoids were pretreated in Advanced DMEM supplemented with BSA (0.1%), HEPES (1%), glutamine (1%), B27 (1%), N2 (1%), niacinamide (10 mM), gastrin (10 nM), N-acetylcysteine (1.25 mM), FGF4 (25 ng/mL), HGF (50 ng/mL), and BMP7 (25 ng/mL) for three days.

GelMA (5%, w/v) and sodium alginate (1%, w/v) were respectively dissolved using saline and were filtered through 0.22 μm filter. Pretreated-HOs that dissociated to single cell and HUVEC (3:1) were gently mixed with GelMA for the microencapsulation. Microcapsules were produced using a microfluidic electrospray device and polymerized in 1% CaCl_2_ solution, and cultured in HCM supplemented with BSA (0.1%), B27 (1%), L-ascorbic acid trisodium salt (0.2 mM), Y-27632 (10 μM), N-Acetylcysteine (1.25 mM), HGF (20 ng/ml), dexamethasone (Dex, 0.5 μM), OSM (20 ng/ml) for three days. Thereafter, the cells were maintained in HCM containing BSA (0.1%), B27 (1%), L-ascorbic acid trisodium salt (0.2mM), N-Acetylcysteine (1.25mM), Y-27632 (10 μM), ITS (1%), Dex (0.5 μM), EGF (10 ng/ml), 8-Br-cAMP (100 µM) (Selleck), VK2 (10 µM), LCA (10 µM) for seven days.

**qPCR**

Total mRNA was extracted from SOVHOs using RNAiso Plus. The concentration of mRNA was quantified and standardized to 200 ng/μl. Primers were reverse transcribed utilizing Takara's PrimeScript RT Master Mix, and complementary DNA (cDNA) was subsequently amplified with Takara's Ex Taq DNA polymerase. The primers utilized are listed within **Table S1**.

**Flow cytometry**

Following one-hour incubation in media containing 5 μM Y27632, spheroids of SOVHOs were digested for 10 minutes with 0.125% trypsin and subsequently filtered. Then, the cells were stained using the LIVE/DEAD Fixable NIR Dead Cell Staining Kit (Invitrogen). Data acquisition was performed utilizing the C6 flow cytometer, and subsequent analysis was conducted by FlowJo software.

**Transmission electron microscopy**

Following an overnight incubation in a 10% sucrose solution at 4°C, the SOVHOs were dehydrated using a gradient of ethanol. Then, they were embedded in Epon 812 and Durcupan (Sigma) after being cleared with propylene oxide. Then, the ultramicrotome was used to prepare ultrathin slices, which were subsequently gathered onto copper grids and counterstained. Visualization was conducted using TEM (JEOL, Japan) operating at 120 kV.

**Transplantation of SOVHOs**

Approval for this research was granted by The Affiliated Drum Tower Hospital of Nanjing University Medical School (Ethical approval No. 20230401). Rats were subjected to overnight fasting and subsequently administered (D-Gal at a dosage of 600 mg/kg to induce ALF model To implant into the liver parenchyma in vivo, SOVHOs-loaded microcapsules were suspended in saline at a 1:1 ratio and injected directly into the left lower lobe of the rat liver using syringes fitted with 22-gauge needles.

**Histology and immunofluorescence staining**

Liver tissue samples were fixed in 4% neutral paraformaldehyde solution. Following dehydration and embedding in paraffin, the samples were subsequently sectioned into 7 μm. H&E staining and immunofluorescence staining were subsequently applied to these sections. The samples were deparaffinized, rehydrated, and subjected to heat treatment in a citrate buffer for 20 minutes in a microwave oven. The sections were blocked with 5% serum after cleaning with PBS. Primary antibodies were diluted accordingly and applied to the slides for overnight incubation. After washing with TBST, fluorescent secondary antibodies were employed to detect the primary antibody on the slides. Finally, DAPI staining was performed to visualize the cell nuclei.

**Blood biochemical analysis**

On day 7 after transplantation, blood samples were collected for biochemical analysis through the abdominal aorta. ELISA kits (Servicebio) were employed to quantify serum levels of ALB, urea, ALT, and AST.

**Statistical analysis**

The data were presented as mean ± standard deviation. The data showed in Fig. 4b, d-e, S1, S3, S7-8 were conducted using Student's t-test with OriginPro 2024 (64-bit) software (v.10.1.0.178). The data showed in Fig. 6c-j, S6b, d, S13 were analysed using one-way ANOVA with GraphPad Prism software (v.8.0.2). The significance levels are defined as follows: **P* < 0.05; ***P* < 0.01; ****P* < 0.001; *****P* < 0.0001. All statistical analyses were performed using GraphPad Prism 5. Data from at least three independent experiments were included for each statistical evaluation.

**
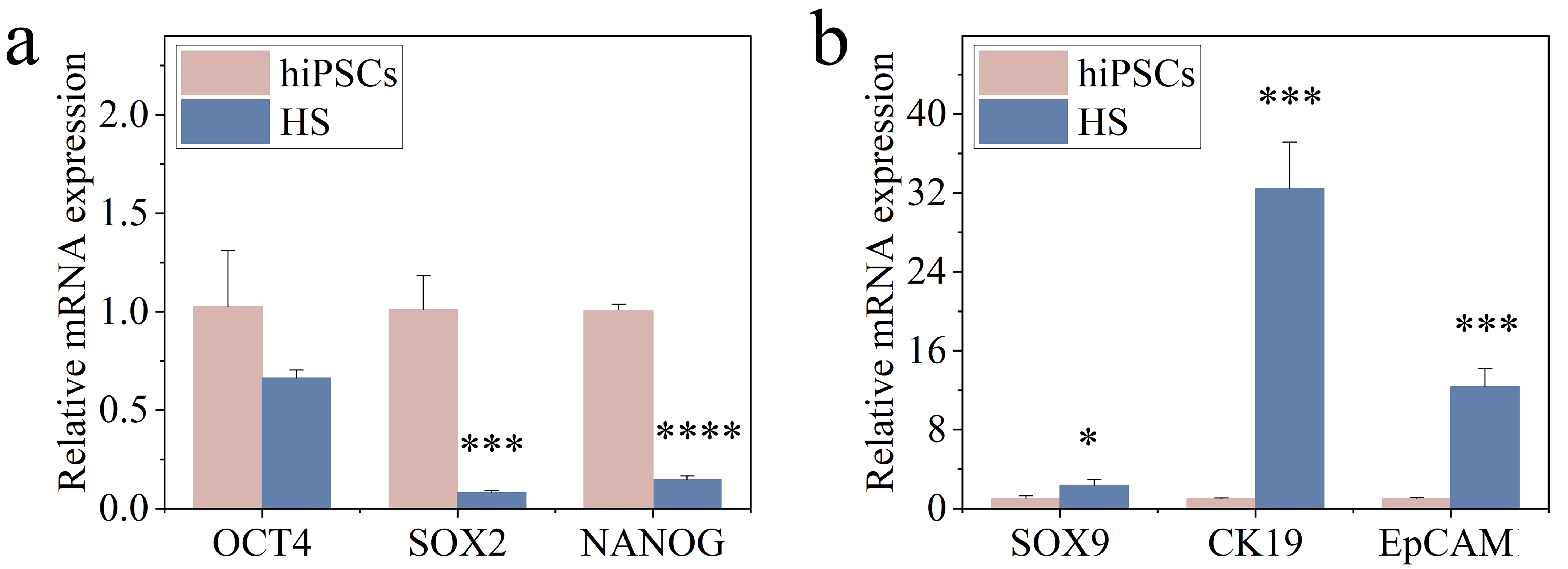
**

**Fig. S1** Expression of pluripotency and hepatic stem cell markers in hiPSCs and

HS was determined by qPCR (n = 3 per group). **P* < 0.05, ****P* < 0.001, *****P* < 0.0001.


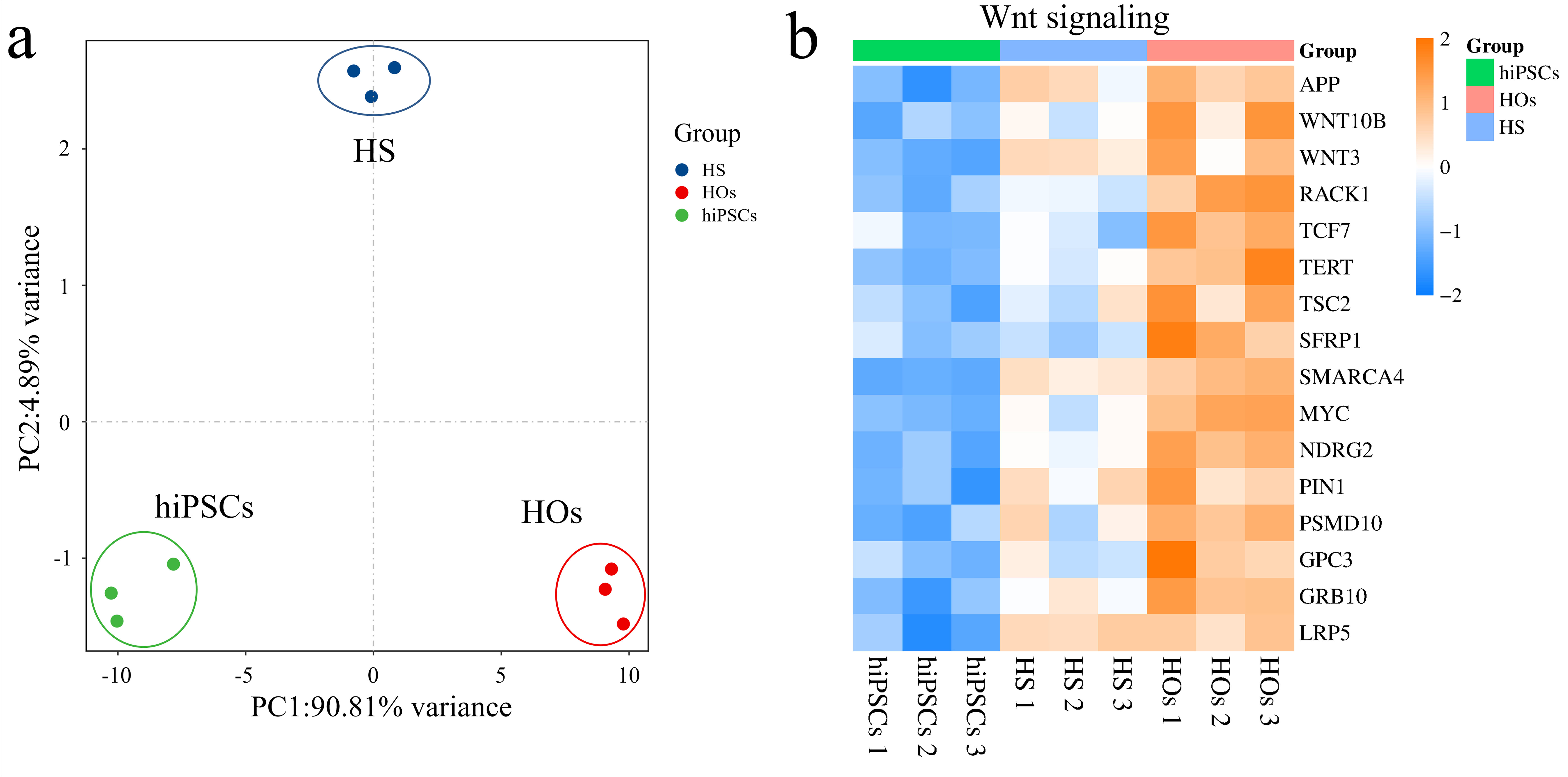


**Fig. S2** (a) Principal component analysis of hiPSCs, HS cells and HOs based on global gene expression profiles. (b) Heatmap showing the expression of genes related to WNT signaling pathway in hiPSCs, HS cells and HOs.


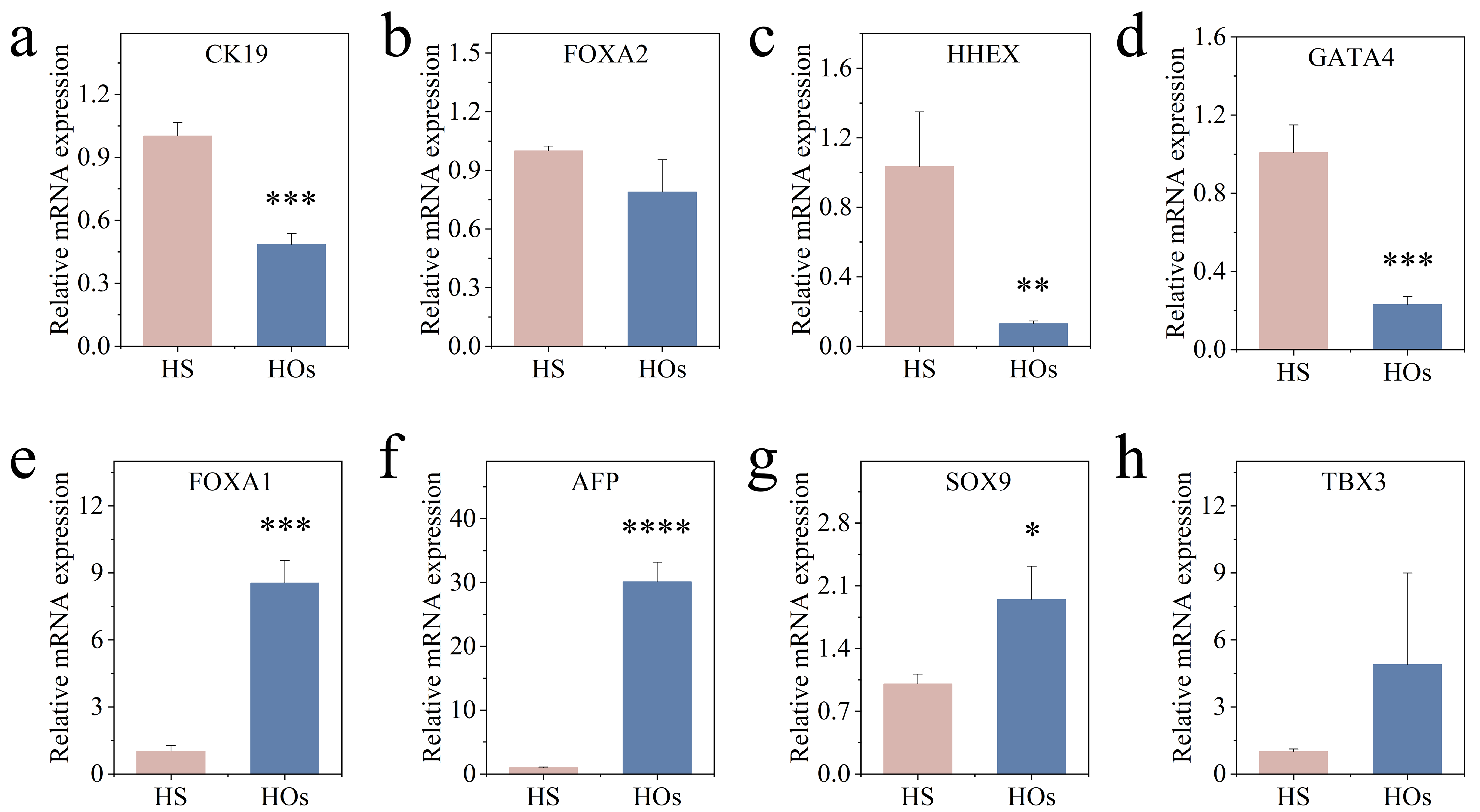


**Fig. S3** qPCR analysis of the expression of markers related early liver development in HS and HOs groups (n = 3 per group). **P* < 0.05, ***P* < 0.01, ****P* < 0.001, *****P* < 0.0001.


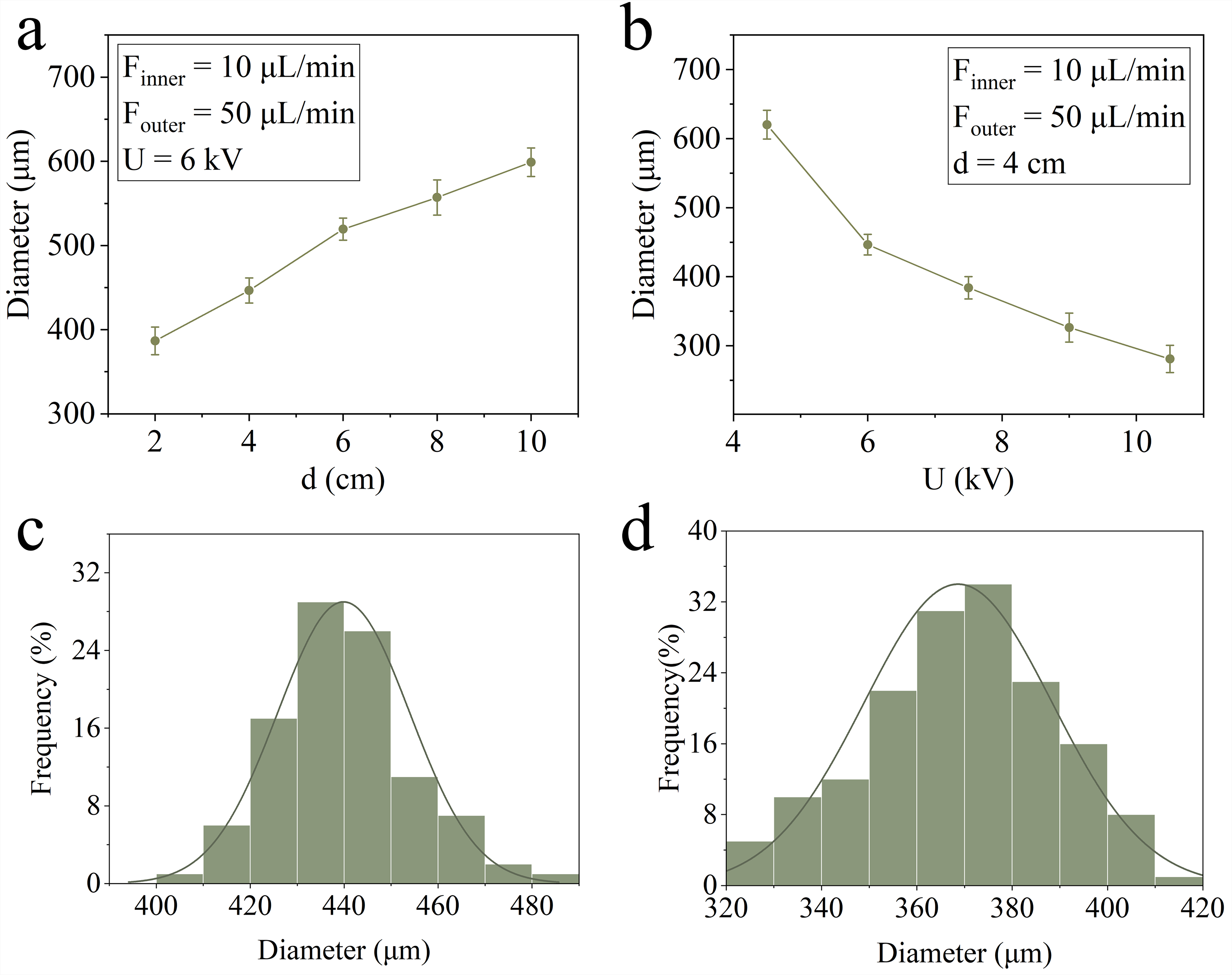


**Fig. S4** (a, b) The relationships between experimental variables (U for voltage, d for collecting distance) and the diameters of the microcapsules. (c, d) Overall size and core size distributions of the GelMA/ALG microcapsules under the parameters listed in the chart.


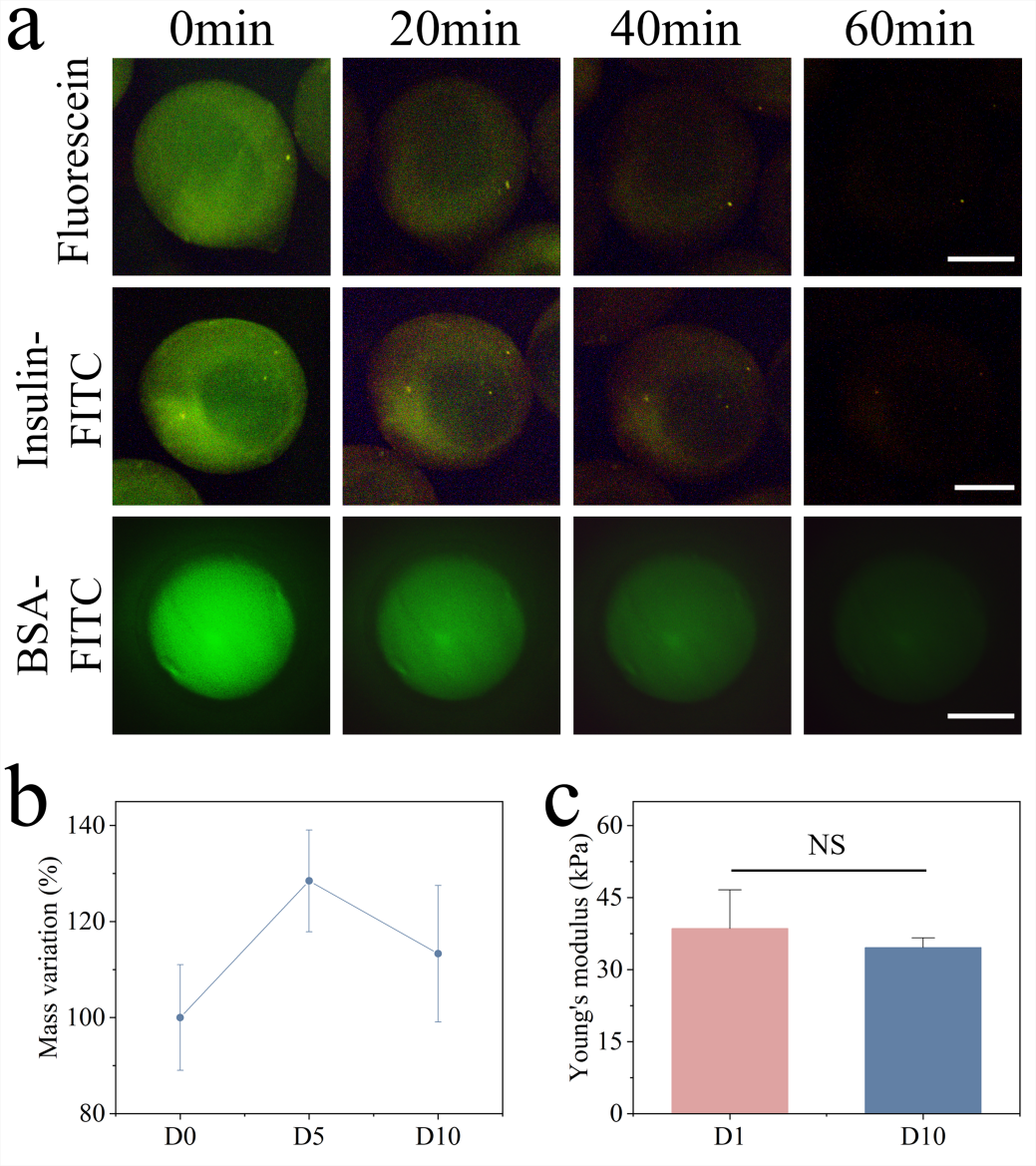


**Fig. S5** (a) Fluorescence images of fluorescein sodium, insulin-FITC, and BSA-FITC diffusion in microcapsules. Scale bar, 200μm. (b) The weight change of the ALG hydrogel within 10 days (n=3). (c) Young’s modulus of the ALG hydrogels after immersion in physiological media for 10 days (n=3)


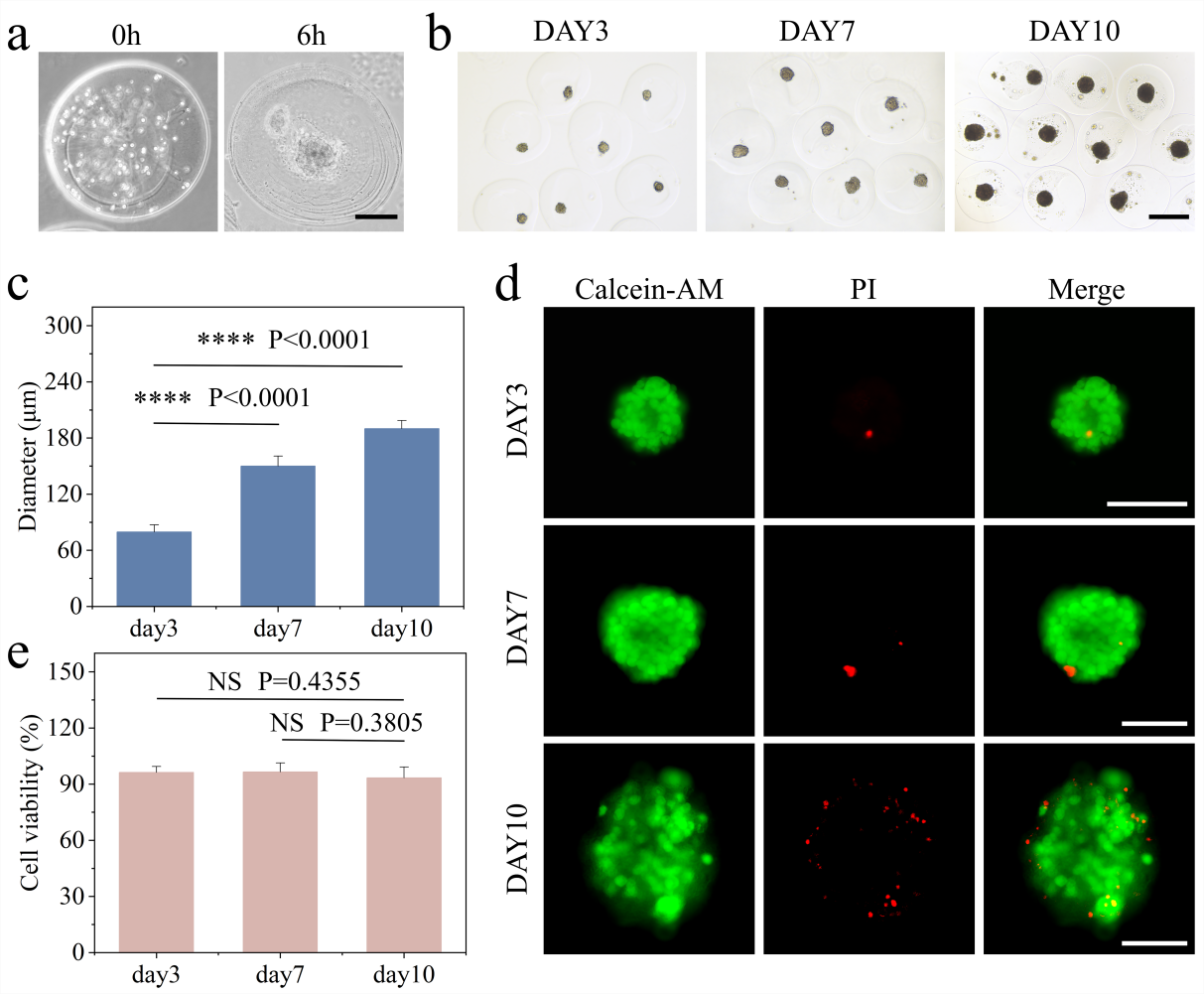


**Fig. S6** (a) Cells self-organizing into spheres in the microcapsule within 6h. (b) The changes in the diameter of SOVHOs during the culture. (c) Quantification of SOVHOs size on day 3, 7 and 10 (n = 30 per group). *****P* < 0.0001. (d) The viability of SOVHOs determined by the calcein-AM/PI staining. (e) Quantification of live cells in SOVHOs on day 3, 7 and 10 (n = 8 per group). Scale bar, 100μm (a,d), 200μm (b). ns, not significant. *****P* < 0.0001.


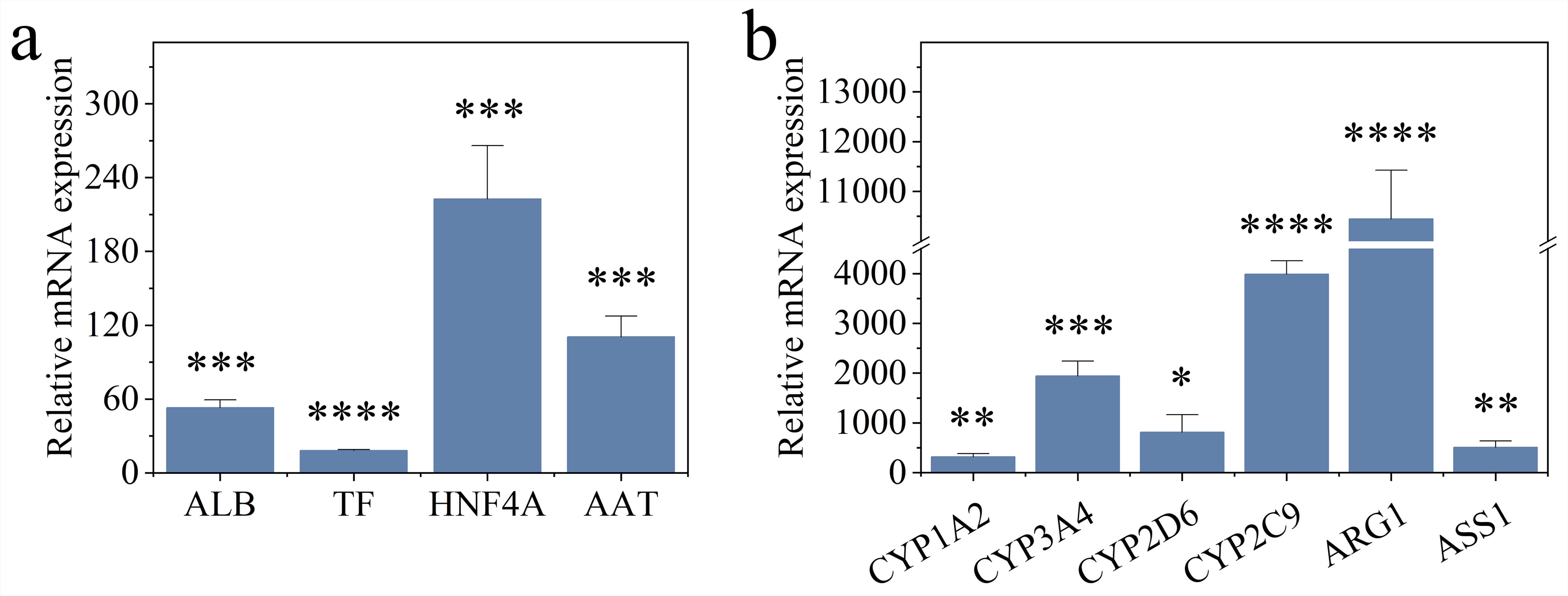


**Fig. S7** qPCR data showing expression of hepatic specific marker in SOVHOs relative to hiPSCs.

**
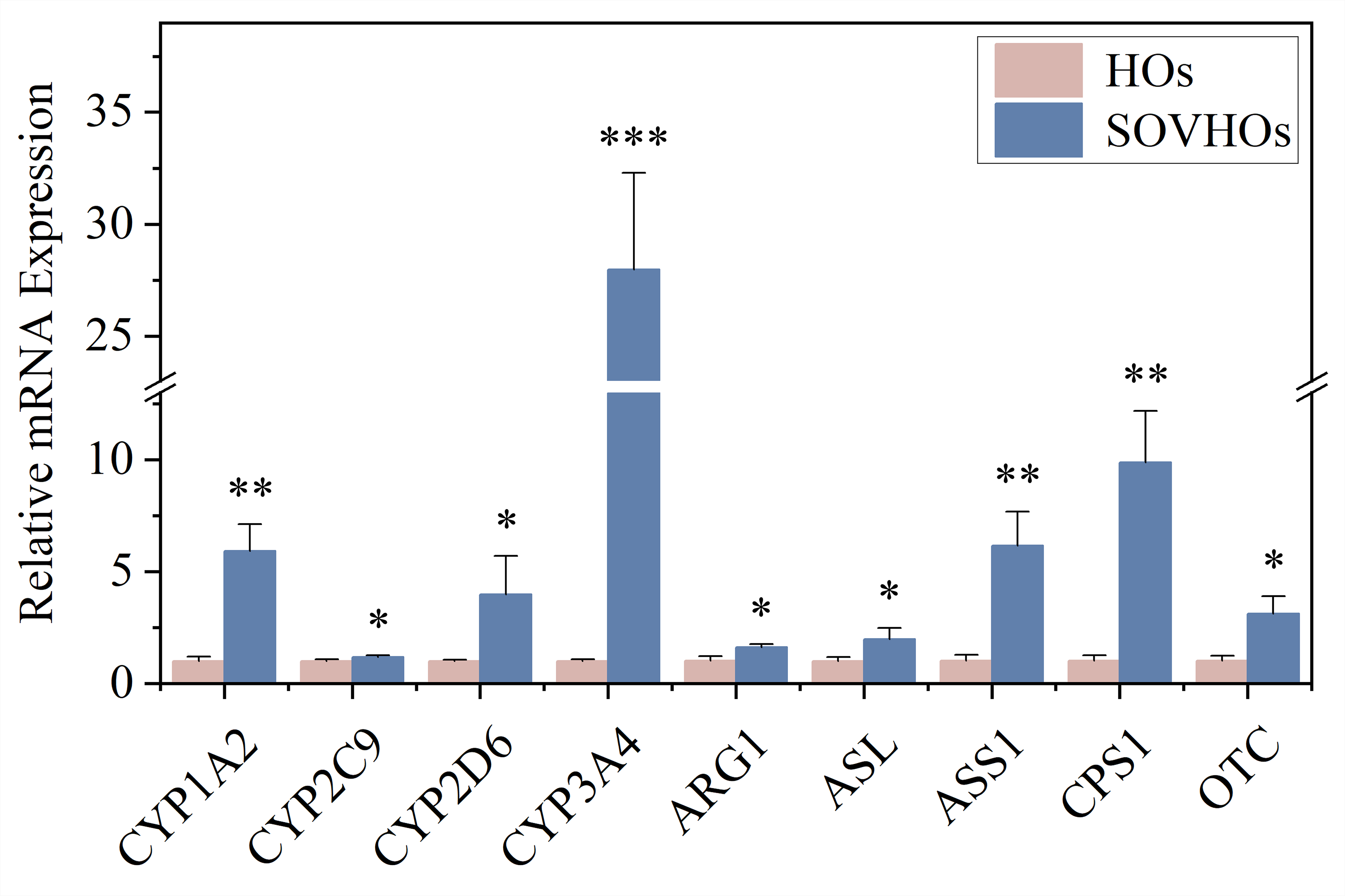
**

**Fig. S8** qRT-PCR analysis for urea cycle-associated genes (CPS1, OTC, ASS1, ASL, ARG1) and genes related to drug metabolism (CYP1A2, CYP2C9, CYP2D6, CYP3A4) in HOs and SOVHOs (n = 3 per group). **P* < 0.05, ***P* < 0.01, ****P* < 0.001.

**
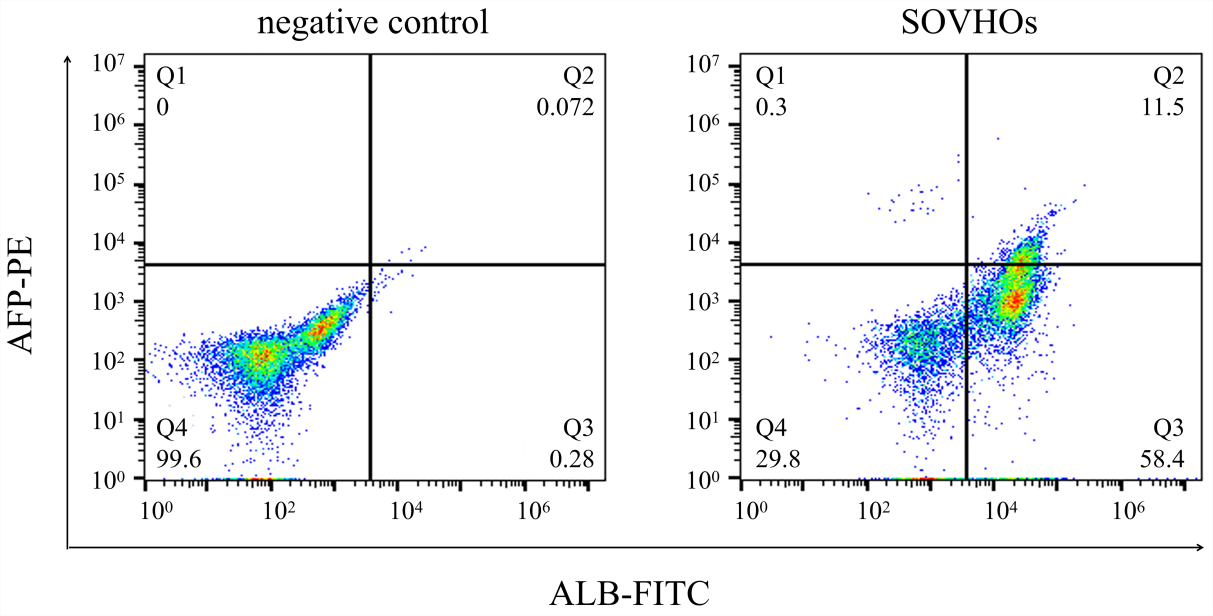
**

**Fig. S9** Flow cytometric analysis showing the proportion of ALB(+) and AFP(+) cells in SOVHOs.

**
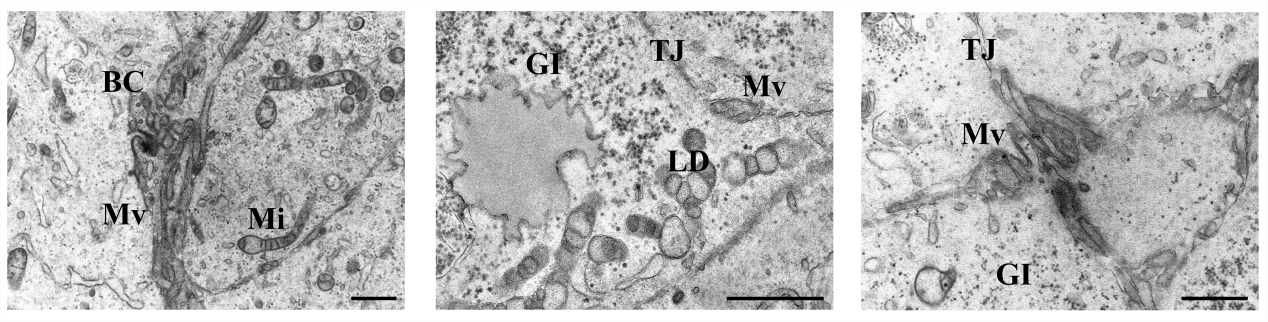
**

**Fig. S10** Ultrastructure of SOVHOs demonstrated the morphological features of mature hepatocytes, including well-developed organelles and intercellular canaliculi. BC: Bile Canaliculi. Mv: Microvilli. Mi: Mitochondria. Gl: Glycogen. LD: Lipid Droplet. TJ: Tight Junction. Scale bar, 1μm.

**
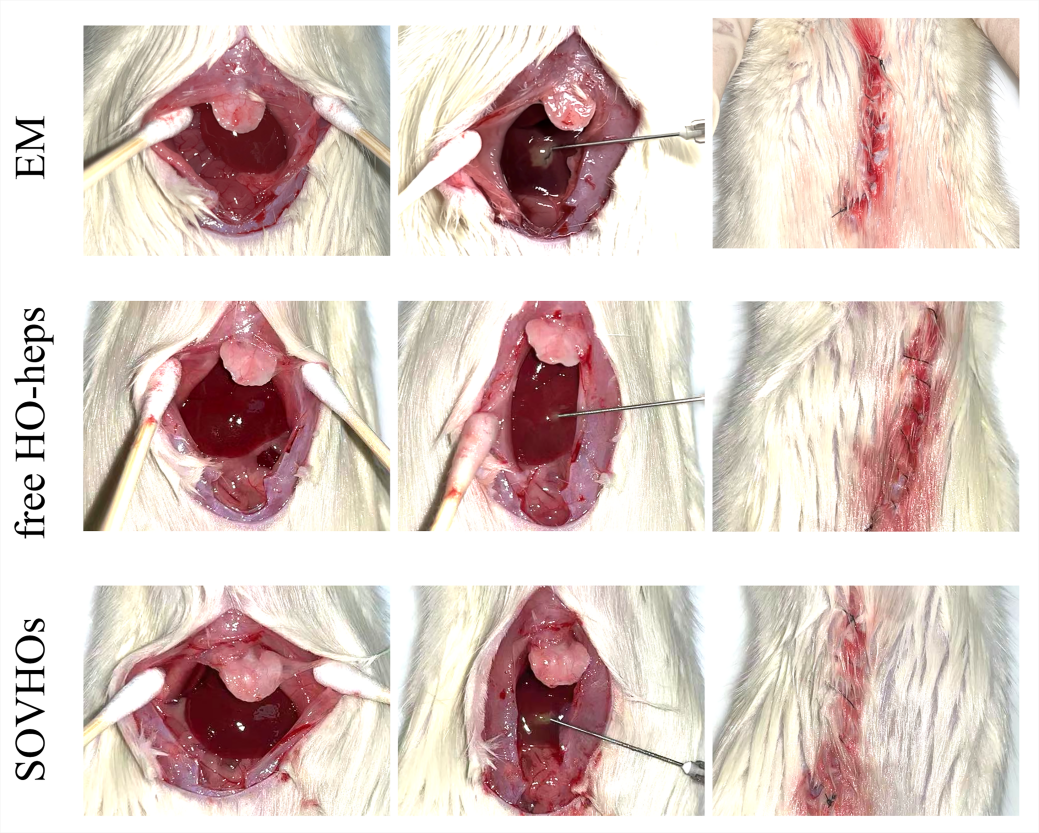
**

**Fig. S11** Images showed the process of transplantation to the liver in vivo.


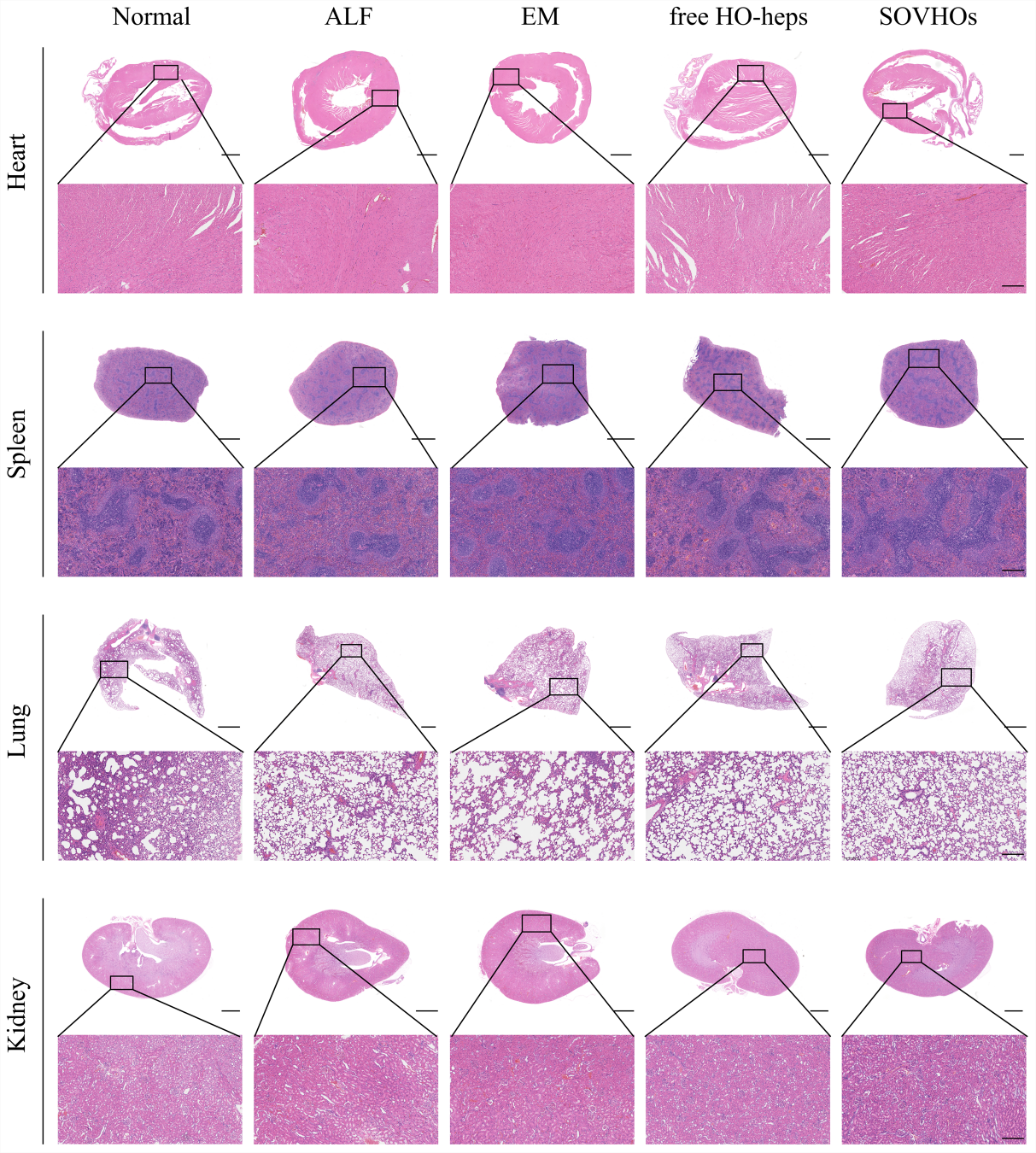


**Fig. S12** H&E staining of heart, spleen, lung and kidney sections from different groups. Scale bar, 2mm (upper line before magnification), 300 μm (lower lines after magnification).


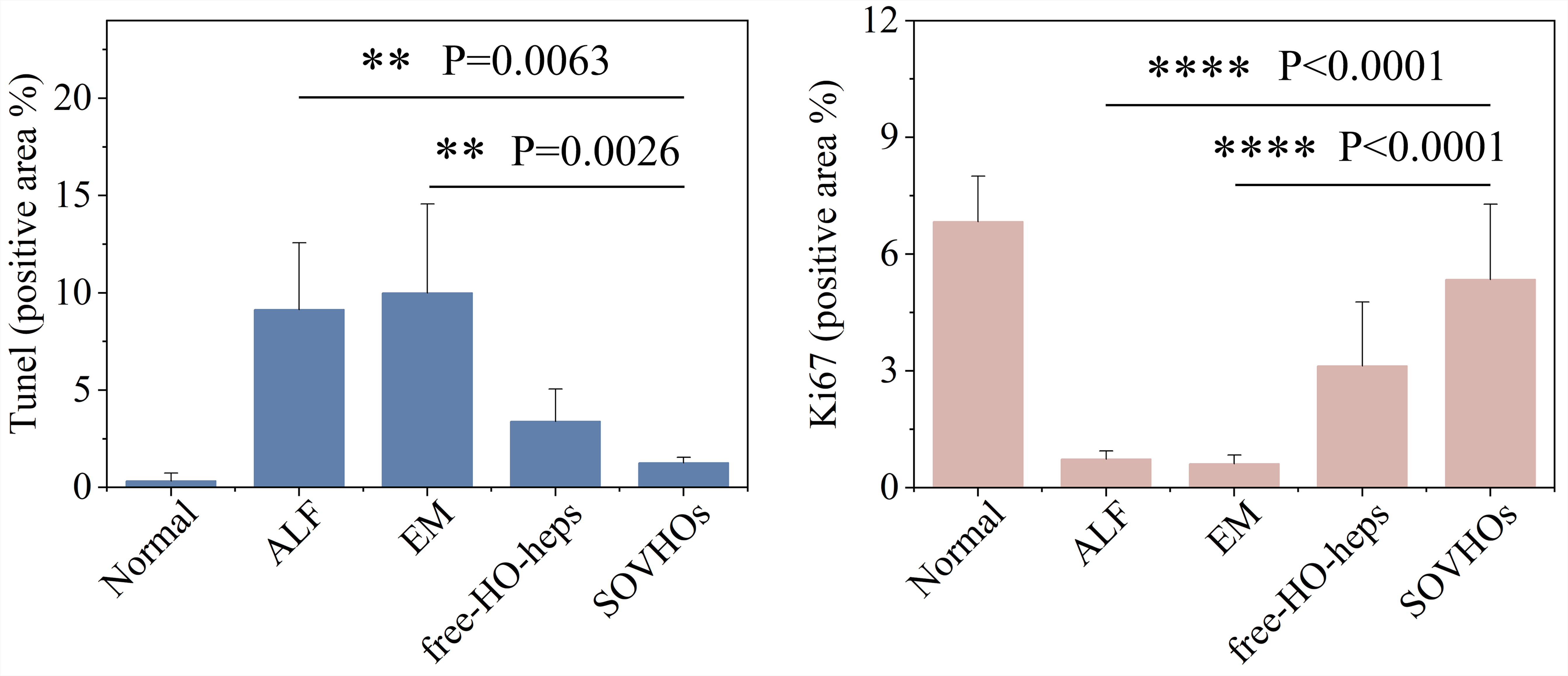


**Fig. S13** Quantification of TUNEL-positive cells and Ki67-positive cells in all groups (n = 3 per group). **P* < 0.05, ***P* < 0.01.

**Table S1 Target gene primer sequences**

| Gene |  | Primers |
| --- | --- | --- |
| OCT | F | CTGGGTTGATCCTCGGACCT |
|  | R | CCATCGGAGTTGCTCTCCA |
| SOX2 | F | TACAGCATGTCCTACTCGCAG |
|  | R | GAGGAAGAGGTAACCACAGGG |
| NANOG | F | TTTGTGGGCCTGAAGAAAACT AGGGCTGTCCTGAATAAGCAG |
|  | R | AGGGCTGTCCTGAATAAGCAG |
| SOX9 | F | AGCGAACGCACATCAAGAC |
|  | R | CTGTAGGCGATCTGTTGGGG |
| CK19 | F | AACGGCGAGCTAGAGGTGA |
|  | R | GGATGGTCGTGTAGTAGTGGC |
| EpCAM | F | AATCGTCAATGCCAGTGTACTT |
|  | R | TCTCATCGCAGTCAGGATCATAA |
| FOXA2 | F | GGAGCAGCTACTATGCAGAGC |
|  | R | CGTGTTCATGCCGTTCATCC |
| HHEX | F | ACGCCCTTTTACATCGAGGAC |
|  | R | CGTGTAGTCGTTCACCGTC |
| GATA4 | F | CGACACCCCAATCTCGATATG |
|  | R | GTTGCACAGATAGTGACCCGT |
| FOXA1 | F | GCAATACTCGCCTTACGGCT |
|  | R | TACACACCTTGGTAGTACGCC |
| AFP | F | CTTTGGGCTGCTCGCTATGA |
|  | R | GCATGTTGATTTAACAAGCTGCT |
| TBX3 | F | GAGGCTAAAGAACTTTGGGATCA |
|  | R | CATTTCGGGGTCGGCCTTA |
| HNF4A | F | CACGGGCAAACACTACGGT |
|  | R | TTGACCTTCGAGTGCTGATCC |
| AAT | F | ATGCTGCCCAGAAGACAGATA |
|  | R | TTGTTGAAGGTTGGGTGATCC |
| TF | F | CCTCCTACCTTGATTGCATCAG |
|  | R | TTTTGACCCATAGAACTCTGCC |
| MRP2 | F | TCTCTCGATACTCTGTGGCAC |
|  | R | CTGGAATCCGTAGGAGATGAAGA |
| ALB | F | GCACAGAATCCTTGGTGAACAG |
|  | R | ATGGAAGGTGAATGTTTCAGCA |
| CYP1A2 | F | CTTCGTAAACCAGTGGCAGG |
|  | R | AGGGCTTGTTAATGGCAGTG |
| CYP2C9 | F | GCCACATGCCCTACACAGATG |
|  | R | TAATGTCACAGGTCACTGCATGG |
| CYP2D6 | F | GTGTCCAACAGGAGATCGACG |
|  | R | CACCTCATGAATCACGGCAGT |
| CYP3A4 | F | GGTGGTGAATGAAACGCTCAG |
|  | R | ACCCCTTTGGGAATGAACATC |
| ARG1 | F | GTGGAAACTTGCATGGACAAC |
|  | R | AATCCTGGCACATCGGGAATC |
| ASL | F | CAGTGGACCCCATCATGGAGA |
|  | R | GGCTTTGCTGCCTTGAACATC |
| ASS1 | F | CTTGGGGCCAAAAAGGTGTTC |
|  | R | GAGGTAGCGGTCCTCATACAG |
| CPS1 | F | AATGAGGTGGGCTTAAAGCAAG |
|  | R | AGTTCCACTCCACAGTTCAGA |
| OTC | F | CGGCCCGTGTATTGTCTAGC |
|  | R | TAGCCAGGGTGTCCAAATCTG |
| GAPDH (Human) | F | AGGTCGGTGTGAACGGATTTG |
|  | R | TGTAGACCATGTAGTTGAGGTCA |
| TNF-α | F | TGAAGTAGTGGCCTGGATTGC |
|  | R | GACATTCCGGGATCCAGTGA |
| IL-6 | F | GCTACCAAACTGGATATAATCAGGA |
|  | R | CCAGGTAGCTATGGTACTCCAGAA |
| IL-1β | F | CCCTGAACTCAACTGTGAAATAGCA |
|  | R | CCCAAGTCAAGGGCTTGGAA |
| iNOS | F | GAGCAAAAAAGGGCAACAC |
|  | R | CGCACTTCTGTCTCTCCAAA |
| GAPDH (Rat) | F | CCCATTCTTCCACCTTTGAT |
|  | R | CAACTGAGGGCCTCTCTCTT |
